# The Treatment of Ulcers of the Cornea in Children

**Published:** 1897-12-25

**Authors:** 


### THE TREATMENT OF ULCERS OF THE CORNEA IN CHILDREN.

Writing on the treatment of idiopathic ulcers of the cornea in children Mr. John Griffiths says that the majority of them will fall under one of the three following headings: (*a*) Symmetrical central ulcers; (*b*) phlyctenular ulcers; (*c*) symmetrical, multiple, vascular ulcers, commonly called "strumous" ulcers. In the central ulcers the centre of each cornea is excavated, as if it had been scooped out; the ulcer is transparent at first, but soon becomes cloudy. It does not, however, become vascular, and rarely extends to perforation. The cause is defective nutrition; the cornea is starved, and a stimulating local treatment is absolutely useless, if not harmful, unless the general nutrition is improved. The child must have some fresh meat every day, and combined with this a vegetable diet. This is the absolutely important point in the treatment.

The phlyctenular ulcers are apt to occur in those who are weakly, not so much from dietetic errors as after the exanthemata or other illness, especially in those who have a tuberculous history. Change, tonics, and wholesome diet are of great use. Locally, calomel may be flecked in with a camel's hair brush once a day, or an ointment of it (gr. 25 to oz. 1) used night and morning; or an ointment of yellow oxide of mercury (gr. 10 to oz. 1) should be introduced between the lids night and morning, and rubbed into the corneal lesion by massage with the eyelid.

When, however, there is pain and photophobia, sedatives are required—atropine and cocaine, which are wisely used in combination. Most phlyctenular ulcers will yield to the use of these drops, and as the symptoms abate the mercurial salts will complete the cure. In the "creeping" variety it may be necessary to touch the growing point of the ulcer with the galvanic cautery.

In the "strumous ulcer" an ointment of atropine, cocaine, and yellow oxide of mercury applied night and morning, with massage, is sufficient in most cases to effect a rapid cure. If the mercury irritates it may be left out until the symptoms subside somewhat. The associated eczema must be treated with boric acid ointment. Other plans are suggested for more severe and obstinate cases, but the above will be found useful in the majority.

